# The effect of alkali concentration on the properties of activated tungsten tailings

**DOI:** 10.1007/s11356-022-24643-9

**Published:** 2022-12-14

**Authors:** Shanmei Li, Kai Shou, Lei Wang, Zhikui Liu

**Affiliations:** Guilin University of Technology, Guilin, South of China 541004 China

**Keywords:** Tungsten tailings, Geopolymer, Alkali-activated, Alkali-solid ratios, Unconfined compressive strength, Microstructure

## Abstract

The 7d unconfined compressive strength tests of alkali-activated tungsten tailings and the microscopic characteristics tests of scanning electron microscope (SEM), Fourier transform infrared spectroscopy (FTIR), and X-ray diffraction (XRD) were conducted to investigate the effect of alkali-solid ratio on the properties of alkali-activated tungsten tailings. The test results indicate that the unconfined compressive strength of alkali-activated tungsten tailings increased with the alkali-solid ratio. However, the strength decreases slightly when the alkali-solid ratio is 12%. The microstructures of the gels generated in the alkali-activated tungsten tailings are affected by the alkali-solid ratio. The details are as follows: the microstructure is honeycomb in low alkali-solid ratio (7%, 8% and 10%), with N-A-S–H as its primary form, and flocculation in high alkali-solid ratio (14% and 15%), mainly in the form of C-A-S–H. When the alkali-solid ratio is at the medium level (12%), the microstructure is a small round bead, and the N-A-S–H is equivalent to the C-A-S–H. The more C-A-S–H content, the greater the strength. This study can provide a scientific basis and technical reference for the resource utilization of tungsten tailings.

## Introduction

In 2004, the Seminar on New Wall Materials for 12 Provinces and Cities in South China held in the Chinese city of Nanchang, pointed out that the production of clay bricks in China destroys 460,000 km^2^ of fertile fields every year, threatening the security of food production. The following year, China issued the document Notice of the General Office of the State Council on Further Promoting the Innovation of Wall Materials and Energy-Saving Buildings, which explicitly prohibited the production of solid clay bricks and developed high-quality new wall materials to replace solid clay bricks. The typical new bricks mainly include sintered porous brick, sintered hollow brick and silicate brick. The former two are still made of clay, which cannot fundamentally change the situation of farmland destruction. Therefore, the research on the silicate brick has more critical practical significance. In recent years, the production of silicate bricks by alkali-activated tailing slag has become one of the research hotspots (Feng et al. 2021; Zhang et al. 2021).

Geopolymer are inorganic cementifying materials formed by active silica-aluminum compounds activated by strong alkali. The concept was put forward by French scientist Joseph Davidovits (Nguyen et al. 2020) in 1978. Geopolymer is a kind of inorganic nonmetallic material composed of the silicon-oxygen tetrahedron and aluminum oxygen octahedron. The polymerization process includes the structural depolymerization of Si–O-Si–O bond and Si–O-Al-O bond to form [SiO_4_] and [AlO_4_] monomer, ion cluster or oligomer, and then polycondensation to form polymer structure (Deng et al. 2016; Aredes et al. 2015; Granizo et al. 2014; Buchwald et al. 2011). Geopolymer mortar has high early strength, exceptional mechanical properties (Phummiphan et al. 2018; Salimi and Ghorbani 2020; Hossein Rafiean et al. 2020), light unit weight, superior durability, less shrinkage and is not easy to react with aggregates (Ghadir and Ranjbar 2018; Vu et al. 2020; Zhou et al. 2021), which can replace cement as a concrete binder (Nazari and Sanjayan 2015). Compared with ordinary Portland cement, producing the same quality of geopolymer can reduce about 70% of CO_2_ emissions, which is in line with the Chinese green development policy. In recent years, it has also attracted the attention of scholars in related fields. The research of Geopolymer in the resource utilization of construction waste and tail slag has achieved fruitful results. For example, Adil et al. used fly ash and ground blast furnace slag as aluminosilicate sources to prepare geopolymer concrete with strength up to 60 MPa (Adil et al. 2021). Ahmari et al. considered the physical and mechanical properties of copper tailings in Tucson, Arizona, under the conditions of NaOH concentration, moisture content, molding pressure and curing temperature (Ahmari and Zhang 2012); Fang et al. analyzed the compressive strength of copper tailings with different lime-sand ratios, the results show that copper tailings with low SiO_2_ content can be used to produce autoclaved lime-sand bricks of grade MU15 (Fang et al. 2011); Peurmal et al. took sodium silicate, sodium sulfate, and sodium carbonate as activators to explore the effects of different alkali sources on the properties of alkali-activated silicate tailings (Perumal et al. 2021); Zhang et al. used sodium hydroxide solution as the alkaline reagent to figure out the effects of fly ash content, NaOH concentration and curing time on the geological polymerization of copper tailings (Zhang et al. 2011); Shahedan et al. used fly ash as precursor material, the sodium hydroxide and sodium silicate mixture as the alkaline activator to prepare new geopolymer concrete. The strength of the geopolymer concrete prepared is 52.58 MPa for 28 days, 54.92 MPa for 60 days and 65.25 MPa for 90 days (Shahedan et al. 2021); Wang et al. studied the compressive strength and hydration performance of alkali-activated fly ash and slag mixture materials based on the different activities of MgO (Wang et al. 2021).

The silicon aluminum compounds in the materials used in these studies are rich and high reactivity, while a large amount of materials with low reactivity of silica aluminum compounds are less studied. The tungsten tailings are one of the most representative these materials. The tungsten resources are rich in China, which grade is low, and the tailings rate is more than 95% (Huang et al. 2011). Tungsten tailings in China are characterized by fine particle size and grave slime, some of which contain heavy metals such as copper and cadmium. At present, some scholars have used different hydrometallurgical techniques to study the recovery of useful components from tungsten tailings, but the recovery efficiency is low and the cost is high (Jane et al. 2021). Accumulation is the primary treatment method of tungsten tailings slag, which occupies much land and causes water pollution, debris flow, landslide, dam cracking, and other geological disasters. It is necessary to study the treatment of tungsten tailings.

Alkali-activated high active tailings have achieved remarkable results. As well as we know, the silicon in tungsten tailings is mainly in the form of quartz with a low reaction activity, and the content of aluminum is low. The formation mechanism of geopolymer in tungsten tailing which is low active materials is unclear. In order to achieve this goal, the mixtures are prepared, which include tungsten tailings as the main component, metakaolin, and commercial geopolymer for increasing the content of aluminum. At the same time, the high temperature was adopted to enhance the reactivity of Si in the specimens. Seven specimens with different alkali-solid ratios (NaOH mass/solid mass) were pressed to study the potential of forming alkali-activated materials by the silicon aluminum compounds with low activity. The unconfined compressive strength of the specimen was tested by uniaxial compression testing. Combined with the microscopic characteristics tested by XRD, SEM, and FTIR technology, the strength and the action mechanism of tungsten tailings activated by different concentration of NaOH solution were analyzed.

## Test materials and methods

### Test materials

**Fig. 1:**
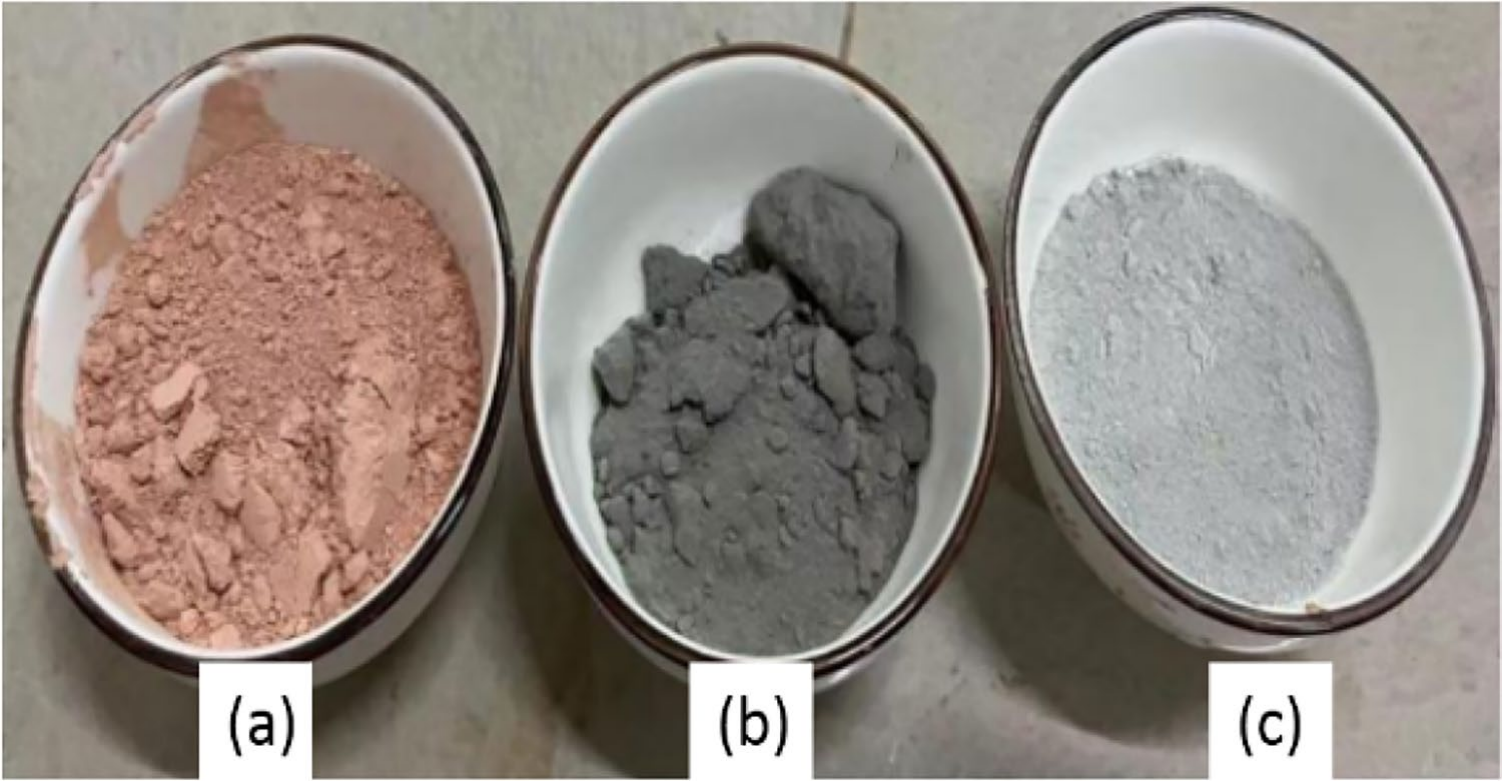
The raw materials of (**a**) metakaolin, (**b**) geopolymer, and (**c**) tungsten tailings

**Fig. 2:**
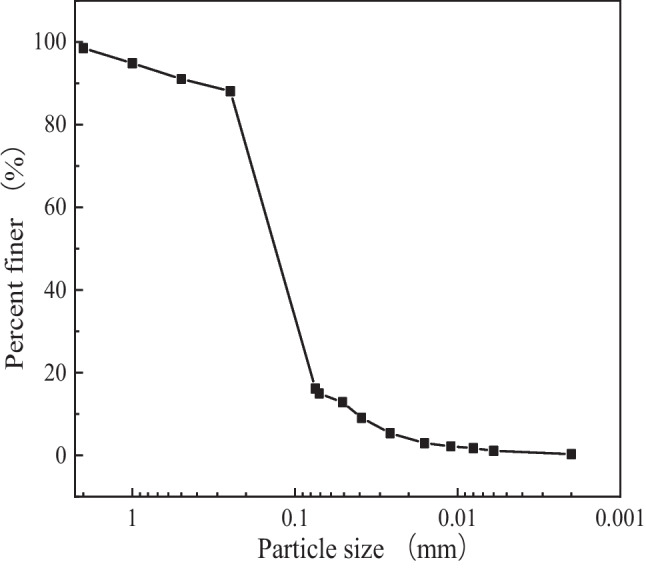
Particle grading curve of tungsten tailings

The materials used in this study include tungsten tailings, metakaolin, and geopolymer, as shown in Fig. 1. The tungsten tailings are taken from Guilin, a city in southern China. The slag is a grey-white powder with a specific gravity of 2.76, and the particles are fine and uniform with 71% with a size of 0.075 ~ 0.25 mm, classified as fine sand, and the particle grading curve is shown in Fig. 2. The sodium hydroxide is of analytical purity, and the content is greater than 96%.

**Fig. 3:**
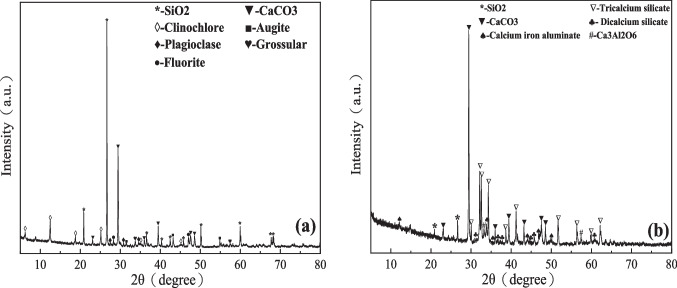
XRD patterns of (**a**) tungsten tailings and (**b**) geopolymer

The XRD patterns of tungsten tailings and geopolymer are shown in Fig. 3. The tungsten tailings are mainly composed of quartz, calcium carbonate, augite, clinochlore, plagioclase, grossular, and fluorite, among which the relative peak strength of the characteristic peaks of quartz (26.8° 2θ) is the strongest, followed by calcium carbonate, which is about 29.8° 2θ, and the relative peak strength of the characteristic peaks of other substances is weaker. The main mineral components of the geopolymer are calcium carbonate and tricalcium silicate, among which the relative peak intensity of the characteristic peaks of calcium carbonate is the strongest, followed by quartz, and the relative peak intensity of other substances is weaker.

The specific composition and content of tungsten tailings and geopolymer are shown in Tables 1 and 2.

**Table 1:** Main mineral composition of tungsten tailings

Mineral composition	Content (%)	Mineral composition	Content (%)
Quartz	43	Grossular	7
Plagioclase	2	Augite	6
Microcline	3	Amphibole	1
Calcite	31	Total clay	5
Fluorite	1

**Table 2:** Main mineral composition of geopolymer

Mineral composition	Content (%)	Mineral composition	Content (%)
Calcite	49	Quartz	5
Tricalcium silicate	28	Calcium iron aluminate	6
Dicalcium silicate	8	Ca_3_Al_2_O_6_	4

The main oxide types of tungsten tailings and metakaolin were tested by fluorescence spectroscopy. The tungsten tailings mainly contained SiO_2_, CaO, and Fe_2_O_3_, accounting for 81.77% of the total oxide composition. The specific components and contents are shown in Table 3. Table 4 shows that the metakaolin mainly contains SiO_2_ and Al_2_O_3_, accounting for 98.08% of the total oxide composition.

**Table 3:** Oxide types in tungsten tailings

Oxide composition	Content (%)	Oxide composition	Content (%)
SiO_2_	40.05	MnO	0.68
CaO	29.30	TiO_2_	0.41
Fe_2_O_3_	12.42	WO_3_	0.30
Al_2_O_3_	6.90	BaO	0.29
MgO	4.33	PbO	0.24
K_2_O	2.12	SnO_2_	0.16
SO_3_	1.67	Cs_2_O	0.08
P_2_O_5_	0.70

**Table 4:** Oxide types in metakaolin

Oxide composition	Content (%)	Oxide composition	Content (%)
SiO_2_	55.06	CaO	0.17
Al_2_O_3_	43.02	MgO	0.06
Fe_2_O_3_	0.76	K_2_O	0.55
TiO_2_	0.24	Na_2_O	0.06

### Specimen preparation and test method

Considering that the specimen could be mixed evenly and the mixture would not be extruded due to excessive pore water pressure during the pressing process, this study adopted a uniform moisture content (19%, 124 g) and solid components (tailing slag, geopolymer and metakaolin) to change the alkali-solid ratio. The unconfined compressive strength of specimens with seven alkali-solid ratios were studied and the specific mix ratio of materials were shown in Table 5. It is undoubted that the strength of alkali activated materials gradually increases with the temperature. However, the rate of increase decrease when the temperature is higher than a certain temperature. For example, Li et al. versified that the strength at 90 °C is slightly higher than at 80 °C (Li et al. 2022). By considering the cost and the engineering utilization requirements, the curing temperature is designed to 80 °C. According to the forming mechanism of the activated materials, the amount of consumed water which dissolute the Si-Al compounds is equal to the amount of produced water in the reaction. In another words, the water has not been consumed in the process of reaction. However, the water evaporates quickly will increase the internal pores of the specimen in the curing process, it results that the strength decreases and to the reaction process ends early. So it is necessary to wrap the specimen with plastic wrap for preventing the water from evaporating quickly. The recent research results show that the strength development of mineral polymeric materials is mainly within 2 to 7 days (Ma et al. 2002a, b), so the specimens in this paper were conserved in oven for 7 days.

**Table 5:** Mixture ratio

Serial number	Alkali-solid ratios (%)	The quality of NaOH (g)	Fixed composition
1	0	0	Slag (586 g) 、Geopolymer (7 g) 、Metakaolin (59 g) 、Distilled water(124 g)
2	7	45.6	
3	8	52.2	
4	10	65.2	
5	12	78.2	
6	14	91.3	
7	15	97.8	

The tungsten tailings were dried in an oven at 105℃, crushed, and screened through 10 mesh for reserve. According to the mix ratio design in Table 5, the 124 g distilled water was first mixed with the sodium hydroxide particles and stirred when preparing the sodium hydroxide solution. After the sodium hydroxide particles were completely dissolved, stopped stirring and cooled the solution to room temperature. Then, weighed 586 g of tailings slag, 7 g of geopolymer, and 59 g of metakaolin, respectively, and mixed them evenly with a JJ-5 cement mortar mixer. Slowly added the cooled sodium hydroxide solution into the solid mixture and continued stirring for 10 min to ensure the uniformity of the mixture. After mixing, filled the mixture in layers into the 70 mm*70 mm*120 mm of self-made steel mold, which is in favor of preventing the materials being extruded in the specimen preparation process and facilitating demoulding. After each layer was filled, flattened and scraped, continued to fill. Then used UTM5305 microcomputer controlled electronic universal testing machine to compress the height of the specimen to 70 mm to form it, and the axial compression rate was controlled to 0.1 kN/s. After the compression was completed, the testing machine could not unload the force immediately. It should be kept in the compression state for 2 min to prevent the rebound of the specimen from causing different specimen sizes. Then, the testing machine was returned to zero, took out the specimen, and demoulded with the DTM-150 Electric Demoulding machine, numbered, wrapped with plastic wrap, and placed in the oven at 80℃ for seven days. Two groups of parallel specimens for each ratio were prepared to reduce the test error, and calculated the average value. After curing for 7 days, took out the specimens, cooled to room temperature, and tested the unconfined compressive strength by using the UTM5305 universal testing machine, and the axial compression rate was controlled to be 4 kN/s. After the compressive strength test, took a small number of specimens, ground with an agate mortar and sifted through 2500 mesh. Tested the mineral composition with the PANalytical Empyrean Type X-ray diffractometer at a scanning Angle of 5° to 80°(2θ°), and the scanning speed was 2°/min. Measured the change characteristics of groups in NaOH activated slag by Thermo Nicolet NEXUS 670 Fourier transform infrared Raman spectrometer, with a spectral range of 4000 to 350 cm^−1^. In addition, took a layer of specimens with a volume of about 1 cm^3^ on their surface, cured at 80℃ for 7 days after demolding. Broke the natural section by hand, and carried out the scanning test of the electron microscope by GeminiSEM300 field emission scanning electron microscope, with a working voltage of 5 kV and a magnification of 10,000 times.

## Results and discussion

### Mechanical properties

#### Relationship between alkali concentration and initial setting strength

The solid specimens involved in this study are unsaturated and mainly fine sand particles with high permeability. Therefore, the deformation characteristics of unsaturated soil (Yin and Ling 2007) can be used to explain the compression process of solid specimens in this paper. The compression process is as follows:

- ①-Gas extrusion;
- ②-Water and enclosed gas are compressed;
- ③-Excess pore water pressure is discharged and dissipated;
- ④-Soil particles (agglomerates) are compressed.

**Fig. 4:**
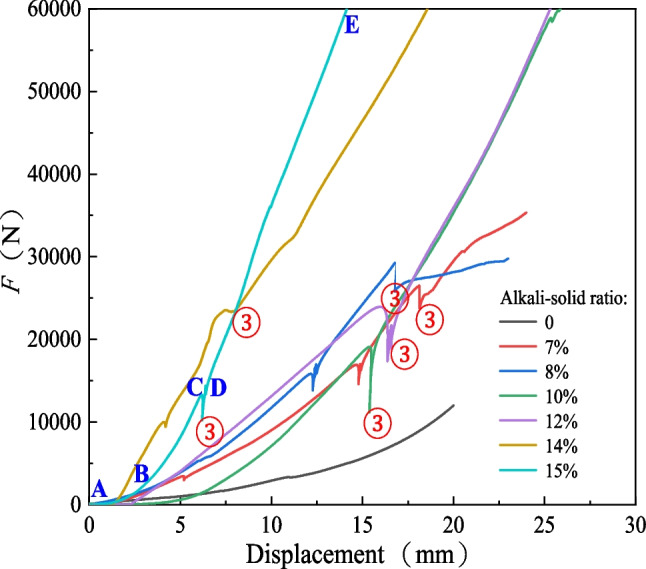
Compression curve of specimen preparation with alkali-solid ratios of 0, 7%, 8%, 10%, 12%, 14% and 15%

Figure 4 shows the slag specimen preparation process's compression curves with different alkali-solid ratios. The compression curve can be divided into four stages. Take the specimens with an alkali-solid ratio of 15% as an example:

- ①-AB section, the gas is extruded from the pores, and the curve is relatively flat;
- ②-BC section, water and enclosed gas in the soil are compressed and the slope of the curve increases;
- ③-CD section, the excess pore water pressure is discharged and dissipated, forming a sharp trough;
- ④-DE section, soild particles (aggregates) are compressed and the slope of the curve is larger.

Solid specimens with smooth drainage channels do not produce excess pore water pressure or require higher pressure to produce. Figure 4 shows that solid specimens of the same dry density but different alkali-solid ratios, the compression displacement corresponding to excess static pore water pressure (CD curve) is significantly different. The law is as follows: 15% < 14% < 10% < 8% < 12% < 7%, when the alkali-solid ratio is 0, there is no CD stage in the specimen preparation process curve, that is, excess pore water pressure does not occur during specimen preparation in this study. In other words, the permeability of the slag mixture can be significantly changed in a short time after the addition of alkali. It can be preliminarily speculated that the reaction between NaOH and the solid mixture will occur in a short time, blocking the drainage channels of soil particles or forming more closed pores.

According to the definition of compression coefficient,

$$\frac{\Delta e}{\Delta p}=\alpha$$

where $\Delta e$ is the change rate of pore ratio; $\Delta p$ is the pressure change rate, the unit is kPa; $\alpha$ is compression coefficient, the unit is 10^4^ × kPa^−1^.

**Fig. 5:**
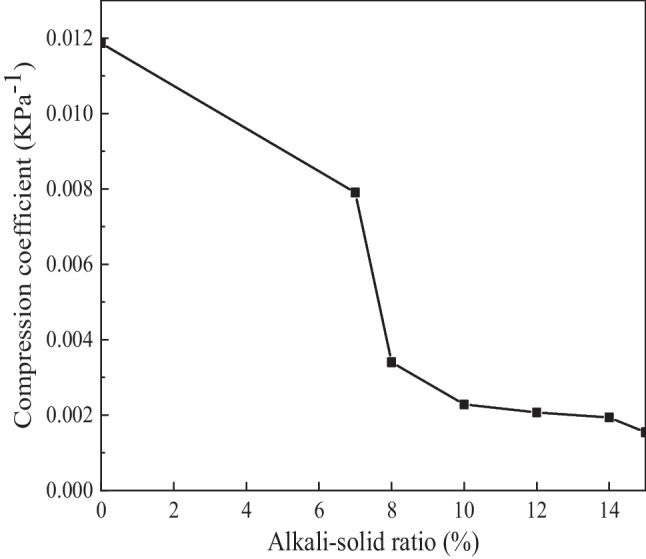
Compression coefficient of specimens with alkali-solid ratios of 0, 7%, 8%, 10%, 12%, 14%, and 15%

Take the straight line segment of the stress-pore ratio curve to calculate the compressive coefficient during the specimen preparation process, as shown in Fig. 5.

Figure 5 shows that as the alkali-solid ratio increases, the compressibility of the soil decreases, which means the greater the alkali-solid ratio, the harder it is to compress the mixture. In other words, the greater the alkali-solid ratio, the greater the force required to compress the specimen with the same dry density.

**Fig. 6:**
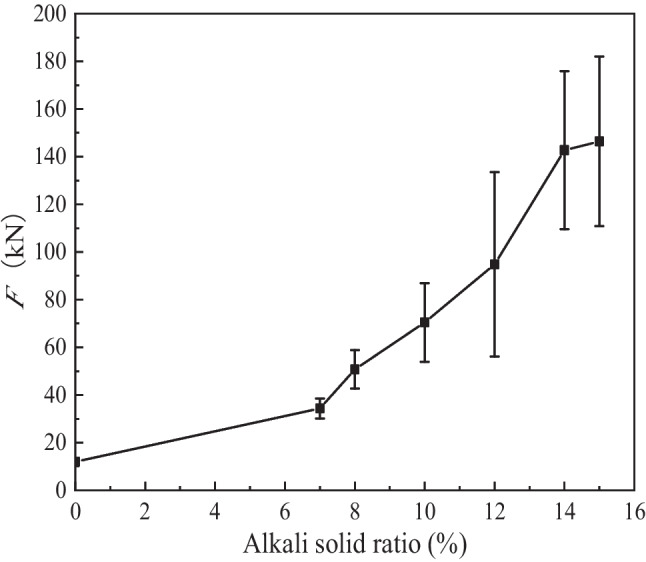
Relationship between forming pressure and alkali-solid ratios of 0, 7%, 8%, 10%, 12%, 14%, and 15%

The relationship between the forming pressure and alkali-solid ratios is shown in Fig. 6.

Figure 6 shows that the average forming pressure increases with the increase of the alkali-solid ratio. When the alkali-solid ratio increases from 0 to 7%, the forming pressure increases by 1.9 times. When the alkali-solid ratio increased from 7 to 15%, the forming pressure increases by 3.3 times. Figures 5 and 6 show that the alkali activated tungsten tailing reacts rapidly, and a certain initial setting strength is obtained during the stirring process of slag and NaOH. The larger the alkali-solid ratio, the higher the initial setting strength of the specimen.

### Relationship between alkali concentration and 7d unconfined compressive strength

**Fig. 7:**
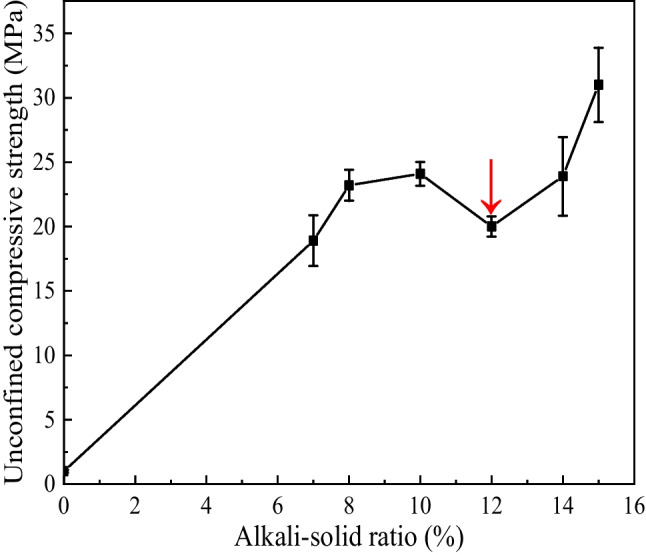
Relationship between unconfined compressive strength and alkali-solid ratios of 0, 7%, 8%, 10%, 12%, 14%, and 15%

After curing for seven days, the unconfined compressive strength of the specimen was tested by a universal testing machine. Figure 7 is the relationship between the average unconfined compressive strength and alkali-solid ratio of three groups of parallel test. It can be seen that after adding sodium hydroxide solution, the 7d unconfined compressive strength of the specimen increases significantly, and the strength increases first, then decreases and finally increases with the increase of alkali-solid ratio. Among them, the alkali-solid ratio of 12% is the particular point. When the alkali-solid ratio is 0, 7%, 10%, 12%, and 15%, the unconfined compressive strengths is 1.0 MPa, 18.9 MPa, 24.1 MPa, 20.0 MPa, and 31.0 MPa, respectively. The relationship between unconfined compressive strength and alkali-solid ratio is as follows: 15% > 14% > 10% > 8% > 12% > 7% > 0.

### Analysis of microscopic characteristics

#### SEM electron microscope characteristics

**Fig. 8:**
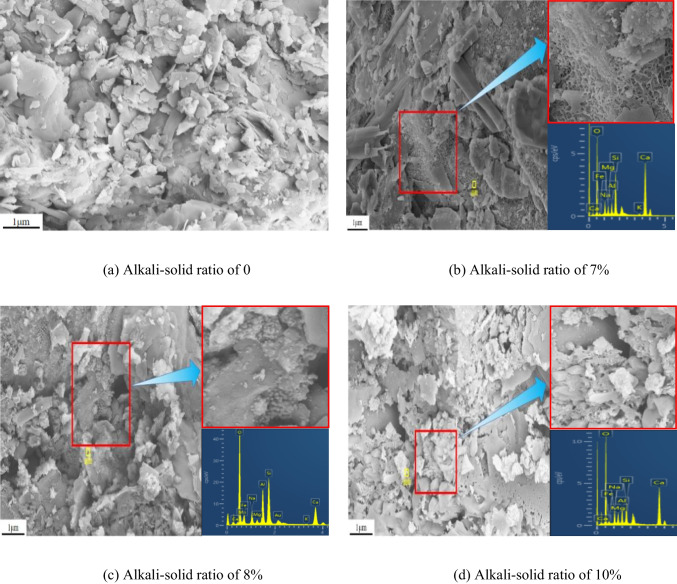

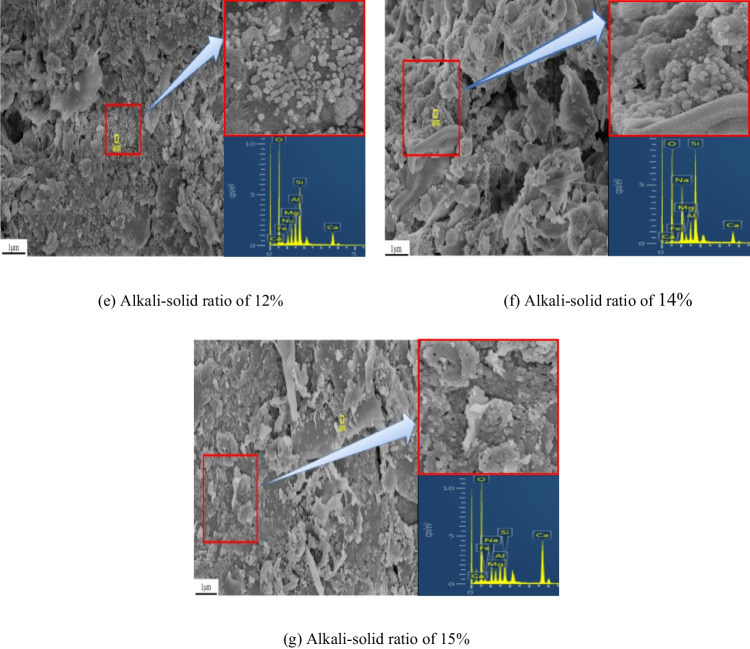
SEM images of tungsten tailings activated by different alkali-solid ratios of (**a**) 0, (**b**) 7%, (**c**) 8%, (**d**) 10%, (**e**) 12%, (**f**) 14%, and (**g**) 15% (× 10,000 times)

Figure 8 shows the microstructure characteristics and energy spectrum of the specimen after curing at 80 °C for 7 days at a magnification of 10,000 times. Among them, (a) to (g) are the alkali-solid ratios of 0, 7%, 8%, 10%, 12%, 14%, and 15% of the specimens. It can be seen from Fig. 8 that under the action of different alkali contents, gel mixtures of N-A-S–H and C-A-S–H are formed in the tungsten tailings. Figure 8(a) shows that when the alkali-solid ratio is 0, the slag mainly has a massive and flake structure, and some smaller clastic particles are irregularly distributed, with a disordered arrangement and loose particles. After adding sodium hydroxide solution (the alkali-solid ratio is 7%), the honeycombed gel substances are generated in the specimen obviously. Honeycombed substances fill between mineral particles and adsorb on the mineral surface, forming a stable large skeleton structure, as shown in Fig. 8(b). When the alkali-solid ratio is 8% and 12%, the specimens take the block and flake structure as the skeleton, and the honeycombed substances disappear, generating a large number of small spherical beads with a diameter of about 50 to 100 nm. The gel substances are distributed on the surface of the large particles and fill between the pores, and the particles are dense, as shown in Fig. 8(c) and (e) respectively. As the alkal-solid ratio increased to 10% and 14%, floccule gel substances appeared, and floccule encapsulated small spherical beads to form large aggregates, as shown in Fig. 8(d) and (f) respectively. When the alkali-solid ratio is 15%, gel increases significantly, and spheroidal gel is distributed on the surface of particles and between pores, with dense particles and no obvious particle boundary, as shown in Fig. 8(g). Gel morphology affects the strength of the specimen. Honeycomb gel has low stability and is easily damaged by adsorption on mineral surfaces. The bead gel has high strength, but it is formed on the surface and pores of minerals and is easy to slide. The flocculated material can encapsulate the mineral particles to form larger and more stable particles with higher strength.

**Fig. 9:**
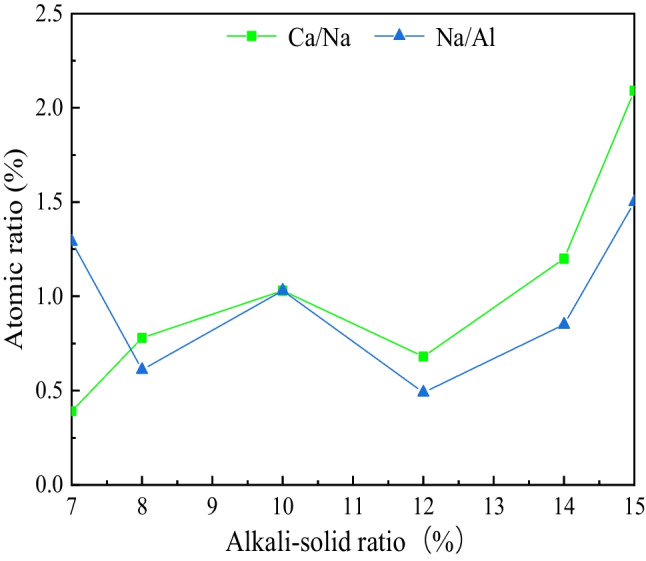
Atomic ratio of gel generated in different alkali-solid ratios of 0, 7%, 8%, 10%, 12%, 14%, and 15%

The atomic ratios of Ca to Na, and Na to Al in the resulting gel was calculated from the energy spectrum, and the results are shown in Fig. 9. With the increase of alkali-solid ratio, Ca/Na increases first, then decreases, and finally increases. The inflection point occurs at 12%. The ratio of Na to Al changes in wavy shape with a small change range. The ranges of Ca/Na and Na/Al in the gel mixtures formed by various alkali-solid ratios are 1.70 and 1.01, and the standard deviations are 0.54 and 0.36.

Figure 9 shows that when the alkali-solid ratio increases from 7 to 15%, the alkali activated slag produces a mixture of N-A-S–H and C-A-S–H gels, which are dominated by N-A-S–H at low alkali-solid ratio and C-A-S–H at high alkali-solid ratio. Combined with Fig. 8, it can be seen that the micromorphology of the gel will be diverse with different Ca/Na. When Ca/Na < 0.4 (the alkali-solid ratio is 7%), the gel is mainly honeycomb. When 0.4 < Ca/Na < 0.8 (the alkali-solid ratios are 8% and 12%), the gel is mainly spherical. When Ca/Na > 0.8 (the alkali-solid ratios are 10%, 14% and 15%), the gel is mainly flocculated. The higher the Ca/Na, the higher the gel content.

### XRD characteristics

**Fig. 10:**
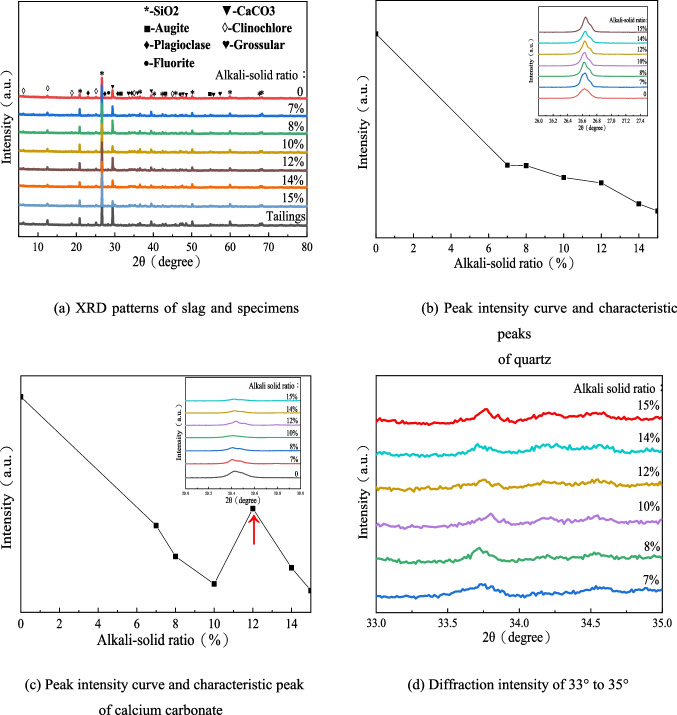
XRD patterns of tungsten tailings activated by different alkali-solid ratios of 0, 7%, 8%, 10%, 12%, 14%, and 15%

Figure 10 shows the X-ray diffraction test patterns of slag and specimens with different alkali-solid ratios (0, 7%, 8%, 10%, 12%, 14%, and 15%). Figure 10(a) shows that the addition of NaOH does not change the peak type of the main crystals in the mixture, but the peak intensity of some minerals, such as quartz and calcium carbonate. The peak intensity of quartz decreases with the increase of the alkali-solid ratio, as shown in Fig. 10(b). The peak intensity of calcium carbonate decreases first, then increases and finally decreases with the increase of alkali-solid ratio. The convex point appears when the alkali-solid ratio is 12%, as shown in Fig. 10(c). After adding sodium hydroxide, metakaolin, and geopolymer, the slag particles will form cementitious substances and the wide peaks, thus reducing the reflection of the rays (Zhang et al. 2013). Meanwhile, the broad peak formed by cementified substances will cover the characteristic peak of other crystals (Nasvi et al. 2012). Therefore, the peak intensity of characteristic peaks of quartz and calcium carbonate will change, as shown in Fig. 10(d), that the dispersion peak of the gelling substance is formed in the range of 33° to 35° in 2θ.

### FTIR characteristics

**Fig. 11:**
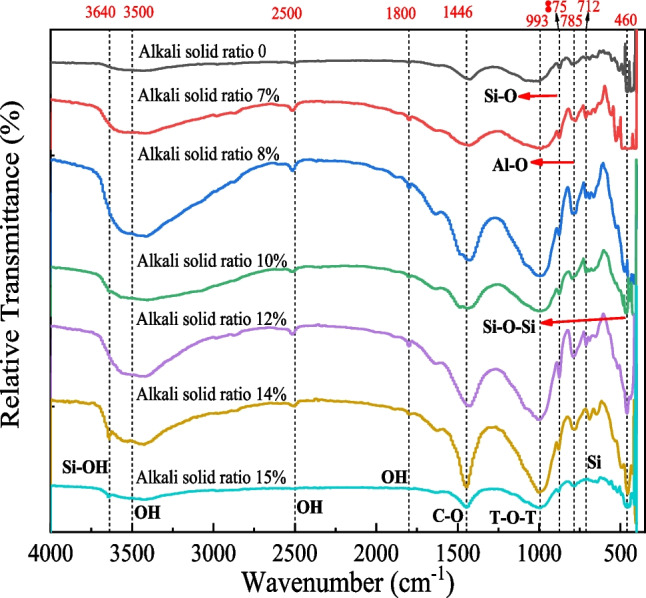
FTIR spectrum of tungsten tailings activated by different alkali-solid ratios of 0, 7%, 8%, 10%, 12%, 14%, and 15%

Figure 11 shows the FTIR spectra of specimens with different alkali-solid ratios. According to the spectrum, the main absorption peaks of specimens with different alkali-solid ratio are 3500 cm^−1^, 2500 cm^−1^, 1800 cm^−1^, 1446 cm^−1^, 993 cm^−1^, 875 cm^−1^, 785 cm^−1^, 712 cm^−1^, and 460 cm^−1^, among which the specimens with alkali-solid ratio of 14% and 15% have one more absorption peak at 3640 cm^−1^. The analysis shows that 3500 cm^−1^, 2500 cm^−1^, and 1800 cm^−1^ are the characteristic absorption bands of hydroxyl, in which the characteristic bands at 3500 cm^−1^ are related to the stretching and bending vibration of the O–H group in hydroxyl produced by silicate hydrolysis, and the characteristic bands at 2500 cm^−1^ and 1800 cm^−1^ are related to the OH bonds in water, Si–OH and Al–OH. The absorption band at 1446 cm^−1^ is related to the stretching vibration of O-C-O, partly due to the CO_3_^2−^ in the specimen, another part is due to the reaction of OH^−^ with CO_2_ in the air to generate CO_3_^2−^. The absorption band at 993 cm^−1^ is related to the asymmetric stretching vibration of the T-O-T group (T = Si or Al), where Si and Al are tetracyclic. The absorption band at 875 cm^−1^ is related to the stretching vibration of Si–O bond and the OH bond in the Si–OH. The absorption band at 785 cm^−1^ is related to the stretching vibration of four coordinated Al-O bonds. The absorption band at 712 cm^−1^ is quartz, indicating that Si in the specimen will not completely dissolve in the geological polymerization reaction. The absorption band at 460 cm^−1^ is related to the bending vibration of Si–O-Si and O-Si–O bonds. However, the absorption bands at 3640 cm^−1^ of specimens with 14% and 15% alkali-solid ratios are isolated non-interacting surface silanol groups, formed by the interaction between fractured Si–O-Si and water. The silanol groups can form a hydrogen bond, exchange hydrogen and transfer electrons with the surrounding substances. Therefore, the strength of specimens with 14% and 15% can increase rapidly, as shown in Fig. 7.

**Fig. 12:**
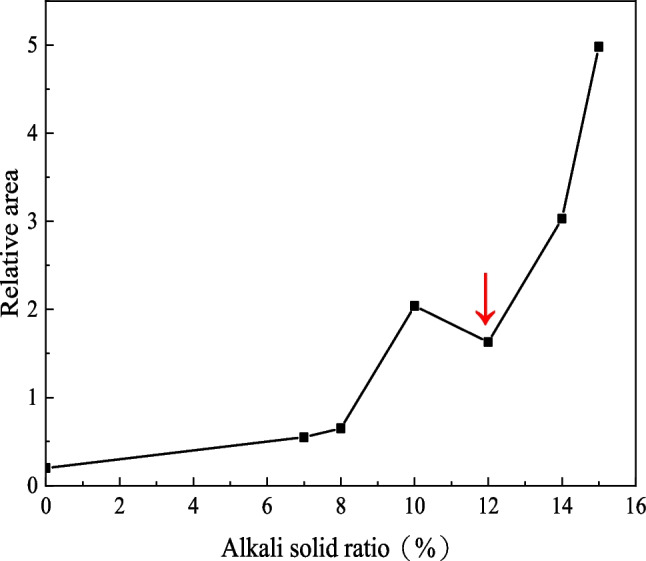
Relative area of peak of FTIR spectrum at 460 cm^−1^ for alkali-solid ratios of 0, 7%, 8%, 10%, 12%, 14%, and 15%

Geopolymerization reaction is susceptible to Si–O radical in the specimen. Since Si–O is related to Si concentration, the peak area at 460 cm^−1^ in the FTIR spectrum can be used as a reference (Aredes et al. 2015). The peak area is a standard method for the quantitative evaluation of geopolymer reaction degree. The peak area at 460 cm^−1^ under different alkali-solid ratios is shown in Fig. 12.

It can be seen from Fig. 12 that with the increase of the alkali-solid ratio, the peak area at 460 cm^−1^ increases first, then decreases and finally increases, which is consistent with the change law of the unconfined compressive strength with the alkali-solid ratio (Fig. 7). The larger the peak area is, the higher the degree of geological polymerization is. As the alkali-solid ratio rises from 0 to 10%, the peak area increases, which means the degree of geological polymerization and the specimen strength enhances. When the alkali-solid ratio is 12%, the peak area decreases, indicating that the degree of geological polymerization is weakened, and the unconfined compressive strength decreases. When the alkali-solid ratio rises from 12 to 15%, the peak area increases rapidly, indicating that the degree of geological polymerization and the specimen strength increases significantly. However, the peak at 0 alkali-solid ratio is mainly due to the addition of a small amount of geopolymer. It can be seen that the peak area is small and the degree of geological polymerization is weak, so the strength of the specimen is the weakest.

## Discussion

According to the XRF test results of tungsten tailings (Table 3), the main minerals are quartz, calcium carbonate, grossular, augite, plagioclase, and microcline. The minimum alkali-solid ratio designed in this study is 7%, the specimen’s moisture content is 19%, and the concentration of NaOH solution is 9.21 mol/L, showing strong alkalinity, and the pH value of the solution is far greater than 12. The active silica mixed with NaOH solution will form sodium silicate (Ma et al. 2002a, b). The chemical reaction is shown in formula (1):

1 $${\mathrm{SiO}}_{2}+2\mathrm{NaOH}\to {\mathrm{Na}}_{2}{\mathrm{SiO}}_{3}+{\mathrm{H}}_{2}\mathrm{O}$$

Metakaolin in the mixture reacts with the NaOH solution. The specific reaction is shown in formula (2):

2 $$nA{l}_{2}{O}_{3}.mSi{O}_{2}+(2n+2m)NaOH\to 2nNaAl{O}_{2}+mN{a}_{2}Si{O}_{3}+(n+m){H}_{2}O$$

The depolymerized Si and Al monomers undergo polymerization, and the specific reaction is shown in formula (3):

3 $$xSi{O}_{3}{}^{2-}+yAl{O}_{2}{}^{-}+nO{H}^{-}+mN{a}^{+}+z{H}_{2}O\to \left(N-A-S-H\right)$$

When the alkali-solid ratio is small, the concentration of OH^−^ in the reaction system is low, and only part of SiO_2_ and Al_2_O_3_ in the slag is dissolved. The reaction only occurs on the surface of solid particles to generate gel flocculation. With the increase of alkali-solid ratio, SiO_3_^2−^ and AlO^2−^ generated by the dissolution of slag increased, and Na reacted with the dissolved SiO^+^_3_^2−^ and AlO^2−^ to form N-A-S–H gel. Simultaneously, Na ions can reduce the electric double-layer thickness and improve the attraction among soil particles (Lang et al. 2021). Therefore, the strength of the specimen increases rapidly after added NaOH (Fig. ^+^7).

When pH > 12, plagioclase will alter to form gel (Zhang and Luo 1993). NaOH mainly reacts with plagioclase as follows:

4 $$CaA{l}_{2}S{i}_{3}{O}_{3}+NaOH\to N{a}_{2}A{l}_{2}S{i}_{3}{O}_{3}+Ca{(OH)}_{2}$$

5 $$Si{O}_{3}{}^{2-}+C{a}^{2+}\to CaSi{O}_{3}$$

6 $$C{O}_{3}{}^{2-}+C{a}^{2+}\to CaC{O}_{3}$$

7 $$xSi{O}_{3}{}^{2-}+yAl{O}_{2}{}^{-}+nO{H}^{-}+mC{a}^{2+}+z{H}_{2}O\to \left(C-A-S-H\right)$$

The dissolved Ca^2+^ reacts with SiO_3_^2−^ and CO_3_^2−^ to generate CaSiO_3_ and CaCO_3_. Excess OH^−^ will catalyze the reaction of formula (7) to form C-A-S–H gel. Under this condition, the activity of the silicoaluminate enhances, the dissolved SiO_2_ and Al_2_O_3_ increase, and more N-A-S–H and C-A-S–H gels are formed. The gel fills the pores between the particles and increases the specimen's compressive strength. Furthermore, the reaction mainly takes place with the anion and cation pairing under the coulomb electrostatic attraction (Ma et al. 2002a, b). The electronegativity of Ca^2+^ is stronger than that of Na, resulting in the formation of C-A-S–H more easily under the high alkali-solid ratio condition. Based on theoretical analysis, the above reaction (^+^1) to (7) all occurs, and satisfies: when the alkali-solid ratio is low, N-A-S–H is mainly in the gel product, and the chemical reactions (1) to (3) occur mainly; when the alkali-solid ratio is high, the C-A-S–H is dominant in the gel product, and the reactions (4) to (7) occur mainly.

Comparing Figs. 7 and 9, the change of the atomic ratio of Ca/Na in the gel with the alkali-solid ratio is completely consistent with the unconfined compressive strength. It can be seen that the content of C-A-S–H in the gel is a key factor affecting the strength of the specimen, which is consistent with the research results of Zhang and Luo (1993).

Besides, this means the content of N-A-S–H is stable in a certain range, and Na/Al varies within the range of 0.5–1.5, which presents a dynamic equilibrium in the specimen.

The reduction of specimen strength may be due to the following aspects: ($\mathrm{I}$) Na ions can rapidly exchange with the other cations during the reaction process (Lang et al. 2021). If OH^+^^−^ concentration is certain, N-A-S–H gels are rapidly generated and coated on the slag surface to prevent C-A-S–H gels formation (Ma et al. 2002a, b and Li et al. 2022). It is not conducive to the improvement of the total gel content that the dissolved Ca^2+^ cations generate calcium carbonate instead of C-A-S–H gels. In fact, the relative content of calcium carbonate in the mixture increases (see Fig. 10 C); ($\mathrm{II}$) The Ca^2+^ cations contribute a lot to stimulate the formation of silicate or polysilicate aluminate networks and harden of the matrix phase. Both of these two behavior enhance the polymer (Ma et al. 2002a, b; Phair and Van Deventer 2001). It results that the strength of C-A-S–H gels is stronger than that of N-A-S–H gels. The higher the content of the C-A-S–H gels, the greater the unconfined compressive strength of the specimens.

## Conclusion

This paper adopts the unconfined compressive strength test to study the mechanical properties and microstructure of improved tailings slag with different alkali-solid ratios when the moisture content is 19%. The micro mechanism of improved tailings slag strength is analyzed by microscopic tests such as SEM and XRD, and the following main conclusions are obtained:

1. The unconfined compressive strength of the tungsten tailings increases first, then decreases and finally increases with the increase of the alkali-solid ratio;
2. The alkali-solid ratio affects the type of gel. The low alkali-solid ratio (7%, 8%, and 10%) is dominated by N-A-S–H; the high alkali-solid ratio (14% and 15%) is dominated by C-A-S–H; the medium alkali-solid ratio (12%), the two are equivalent. The contribution of C-A-S–H to the strength is greater than that of N-A-S–H;
3. The micromorphologies of N-A-S–H and C-A-S–H gel mixtures depend on Ca/Na. Ca/Na < 0.4, the gel is honeycomb; 0.4 < Ca/Na < 0.8, the gel is round like; Ca/Na > 0.8, the gel is flocculent.
4. The strength of alkali activated tungsten tailings consists of initial setting strength and curing strength. The initial setting strength is formed in the specimen preparation process and affects the early strength of the material; the curing strength is formed in the curing process of the specimen. The initial setting strength and curing strength determine the total strength of the material jointly. In this study, the minimum unconfined compressive strength of the specimen is 16.7 MPa and the maximum is 34.3 MPa, which meets the requirements of building wall brick MU15.

## Data Availability

All data, models, and code generated or used during the study appear in the submitted article.
